# Assessment of the SmartJournal Intervention for Improved Oral Care in Nursing Homes: Protocol for a Cluster Randomized Controlled Trial

**DOI:** 10.2196/46926

**Published:** 2023-10-06

**Authors:** Elisabeth Lind Melbye, Vibeke Hervik Bull, Kristin Søllesvik Hidle

**Affiliations:** 1 Research Department Oral Health Centre of Expertise Rogaland Stavanger Norway

**Keywords:** cluster randomized controlled trial, digital tool, gerodontology, intervention, nursing homes, oral health

## Abstract

**Background:**

Poor oral health is common in nursing home residents, and health care professionals report numerous barriers when it comes to the provision of oral care for care-dependent older adults, such as a lack of oral health knowledge and skills, care-resistant behaviors in residents, lack of adequate oral care routines, insufficient systems for documentation of issues related to oral health, a high workload, and unclear responsibilities. An intervention, SmartJournal, has been developed to assist health personnel in preserving older patients’ oral health. SmartJournal is a digital tool with three components: (1) documentation of oral hygiene routines; (2) monthly oral health assessment; and (3) education on geriatric oral care. An updated framework for research on complex interventions has been used to guide the various phases in the development of this intervention. This study focuses on the evaluation phase.

**Objective:**

We aim to assess (1) the effectiveness of SmartJournal use in enhancing nursing home caregivers’ capability, opportunity, motivation, and routines related to oral care; and (2) whether SmartJournal use results in improved oral health for nursing home residents.

**Methods:**

We will use a cluster randomized controlled trial to assess impacts of SmartJournal use as specified under study objectives. Nursing homes located in Rogaland, Norway, will be recruited and randomly assigned to an intervention and a control group. The intervention group will be provided with tablets containing SmartJournal and support from research staff, while the control group will continue with existing oral care routines. Both groups will receive oral hygiene packages. The intervention will last for 12 weeks. Measurements will be performed in both groups at baseline, 3 months, and 9 months and include (1) a survey assessing caregivers’ capability, opportunity, motivation, and routines related to oral care; and (2) assessment of oral health status in residents using mucosal-plaque score as a primary outcome measure. Analyses will include descriptive statistics and statistical techniques to look for changes and differences in the study variables within and between the intervention and control groups throughout the study period. Associations between study variables will also be investigated.

**Results:**

Recruitment of nursing homes for the intervention and control groups was completed in February 2023. Recruitment of individual participants (ie, nursing home residents and caregivers) is ongoing, and baseline measurements are being performed consecutively. Results are expected to be ready for analyses in fall 2024.

**Conclusions:**

We aim to assess the effects of SmartJournal use on oral care and oral health in nursing homes. Results from this work may inform further development and implementation of SmartJournal and similar digital tools aiming to enhance health care services offered to care-dependent people. Finally, SmartJournal may have potential as a learning tool in educational programs for nurses and other health care professionals.

**Trial Registration:**

ClinicalTrials.gov NCT05724043; https://clinicaltrials.gov/study/NCT05724043

**International Registered Report Identifier (IRRID):**

DERR1-10.2196/46926

## Introduction

### Background

Poor oral health is common in frail and care-dependent older adults and includes tooth loss, poor oral hygiene, a high prevalence of dental caries and periodontal disease, defective dental prostheses, hyposalivation, and various oral lesions caused by misfitting dentures or precancerous or cancerous states [1,2]. Alarmingly, oral health is shown to be the most neglected area in Norwegian nursing homes [3], and oral health care is the first task to be downgraded due to a lack of resources [4]. A study by Willumsen et al [5] indicated that more than 40% of Norwegian nursing home residents have unacceptable oral hygiene. Neglect of oral health, resulting in poor oral hygiene, may cause infections and inflammation both locally and systemically. Oral infections have been associated with systemic conditions such as atherosclerosis [6,7], diabetes [8], and aspiration pneumonia [9,10]. Aspiration pneumonia is a highly mortal disease, and a review by Sjøgren et al [11] estimated that 10% of all deaths from pneumonia in nursing homes could have been prevented with improved oral hygiene. Moreover, chewing difficulties as a result of poor dental status have been associated with altered dietary habits, malnutrition, and cognitive decline [2]. Yet, the oral health of care-dependent older adults does not receive the necessary attention [12]. A few decades ago, most older adults were denture wearers. Today, an increasing number of older adults keep their natural teeth, often in combination with complex fixed or removable prostheses [13]. While dentures are easy to manage, more complex prosthetic devices can pose a challenge with regard to handling and cleaning [14]. Thus, the care-dependent older adults have to rely on caregivers to maintain satisfactory oral health [15]. In Norway, nursing home residents (and other care-dependent people) have a statutory right to good oral hygiene and necessary dental treatment [16]. Health personnel are responsible for preserving these rights through daily oral hygiene routines and referral to dental professionals when needed. Research from Sweden and Denmark has shown that about 80% of nursing home residents need assistance with daily oral hygiene practices [15,17]. Thus, it is disturbing that health care professionals report numerous barriers when it comes to the provision of adequate oral care assistance. The most frequently reported barriers are lack of knowledge and skills [18] and care-resistant behaviors (CRB), in particular among patients with dementia [18,19]. A study by Willumsen et al [5] showed that more than 50% of Norwegian nursing home residents with dementia refuse oral care assistance. In cases of frequent CRB, daily oral care may be postponed repeatedly, resulting in poor oral hygiene and an increased risk of oral and systemic infections. Other reported barriers include a lack of adequate oral care routines, a lack of systems for documentation of issues related to oral health [20,21], a high workload, and unclear responsibilities [19].

Various tools have been developed to assist health personnel in providing oral care to older adults. One example is “oral care cards” with illustrated instructions about how to carry out daily oral hygiene routines with individual patients. Another example is “signature lists” to document daily oral care. These tools may function well but have some shortcomings. While oral care cards do not allow for documentation of oral health issues, signature lists do not contain information on individual needs. Moreover, text-based diagnostic tools such as the Revised Oral Assessment Guide (ROAG) [22] and the Oral Health Assessment Tool [23], have been developed to assist caregivers in recognizing oral conditions. However, research indicates that these tools may not be well suited for nondental professionals [24,25]. Other limitations of the above-mentioned tools are that they lack procedures for documentation of deviance related to oral health and that none of them include strategies for handling CRB.

### The SmartJournal Intervention

As a response to the shortcomings of existing tools and to overcome reported barriers to the provision of adequate oral care in nursing homes, an intervention tailored to nursing staff has been developed by researchers at the Oral Health Centre of Expertise Rogaland, Norway, in close collaboration with innovative research and development institutions and dental and health care professionals. An updated framework for research on complex interventions, developed jointly by the United Kingdom’s Medical Research Council and the National Institute for Health Research [26], has been used to guide the various phases of intervention development: an innovation phase (completed), a feasibility study (completed), and an evaluation study (recruitment is ongoing). An overview of the phases is presented in Table 1. This study focuses on the evaluation phase.

**Table 1:** SmartJournal study overview describing aims, methods, and participants for each phase of the study.

Phase	Aims	Methods	Participants
Phase 1: innovation (completed)	Develop an intervention (digital tool) to reduce or overcome barriers related to oral care in nursing homes (theoretical basis: the COM-B^a^ model [27]).	Step 1: collaborative innovation based on current literature, meetings, and workshops with stakeholders resulted in the SmartJournal prototype. Step 2: initial, small-scale functionality tests with the test panel. Step 3: refinement of the tool based on results from functionality tests.	Step 1: practitioners and researchers within dentistry, geriatric nursing, health education, eHealth, and the development of digital learning tools. Step 2: health personnel (n=11) recruited from nursing homes in Rogaland, Norway. Step 3: researchers and engineers.
Phase 2: feasibility study (completed)	Investigate health personnel’s acceptance of and experiences with the SmartJournal prototype in a nursing home setting.	Real-life testing of the SmartJournal prototype in nursing homes. Design: mixed methods, cross-sectional study Qualitative data: thematic analysis of data from semi-structured interviews. Quantitative data: statistical analysis of data from survey questionnaires.	Health personnel were recruited from 3 nursing homes in Rogaland, Norway. Semistructured interviews (n=11). Survey questionnaire (n=86).
Phase 3: evaluation study (in progress)	Evaluate the effectiveness of SmartJournal use on: (1) caregivers’ capability, opportunity, motivation, and routines related to oral care; and (2) residents’ oral health (theoretical basis: the COM-B model [27]).	Step 1: Further refinement of the tool based on results from phase 2. Functionality tests with different test panels. Step 2: 12-week intervention with measurements at baseline, 3 months, and 9 months. Design: Cluster-randomized controlled trial with nursing homes randomly assigned to intervention (SmartJournal use and support) and control group (continue with existing oral care routines). Measures: a survey questionnaire assessing caregivers’ capability, opportunity, motivation, and routines related to oral care. Clinical assessment of residents’ oral health: MPS^b^ [17] and tooth count [28].	Step 1: refinement of the tool, researchers, and engineers. Functionality tests: panels of auxiliary nursing students (n=70) and master students in innovation and knowledge development (n=20). Step 2: a total of 12 nursing homes in Rogaland, Norway: 6 in the intervention and control groups, respectively. Power analyses suggest 360 residents (n=180 in each group). All eligible caregivers are invited to participate.

^a^COM-B: capability, opportunity, motivation model of behavior.

^b^MPS: mucosal-plaque score.

The intervention addresses key behavioral components as postulated by the capability, opportunity, motivation model of behavior (COM-B) [27]. According to the COM-B model, there must be sufficient capability (C), opportunity (O), and motivation (M) present for a given behavior to occur. Hence, this intervention seeks to facilitate oral care in nursing homes by providing strategies to improve caregivers’ capability, opportunity, and motivation for this task. The intervention is constructed as an interactive and easy-to-use app for tablet named SmartJournal. SmartJournal consists of 3 modules. Module 1, “registration of oral hygiene routines,” offers a system for simple checkbox registration of daily oral hygiene routines (ie, whether they have been performed or not). It also includes an open text field for additional comments if relevant (eg, reasons for not performing oral hygiene routines or strategies used to reduce CRB in the patient). This straightforward registration ensures documentation of oral hygiene routines and deviations related to such routines, keeping health personnel informed about potential oral hygiene issues for individual patients. Module 2, “monthly oral health assessment,” offers a system for monitoring the patient’s oral health status through monthly check-ups. In this component, the principles of the text-based tool ROAG are transformed into an image-based, interactive learning and assessment tool. As for ROAG, conditions for 6 oral sites (lips, mucous membranes, tongue, gingiva, teeth, and prosthetic devices, including all forms of removable dentures and fixed prosthodontics) are graded as follows: grade 1=healthy or normal condition, no need for measures; grade 2=pathology, need for measures at a “local level” (ie, at the nursing home, delivered by nursing home personnel); and grade 3=advanced pathology, need for measures delivered by dental professionals (in the nursing home if feasible, at a dental clinic if necessary). SmartJournal contains several validated example pictures for each grade for all 6 oral sites. This part of the tool is also checkbox-based to limit health personnel’s workload related to documentation. Upon completion of the monthly oral health assessment, health personnel receive a summary report with suggestions for tailored preventive or curative measures to be initiated, thus reducing the risk of deteriorated oral health in the patients. Module 3, “electronic learning (e-learning),” offers an easily accessible knowledge base that includes information on geriatric oral health, standard equipment and procedures for daily oral hygiene routines in patients with natural teeth and various prosthetic devices, and oral care for patients with special care needs (including patients with swallowing difficulties, bedridden patients, and patients on palliative care). It also includes detailed information on scientifically documented strategies that may be used to approach patients with dementia who refuse oral care assistance. These strategies are adapted from the interventions Managing Oral Hygiene Using Threat Reduction [29,30] and Mouth Care Without a Battle [31]. The content in this part of the tool is illustrated with photos and figures and includes a video with recommendations on how to handle situations where patients refuse oral care assistance. The acceptability of the SmartJournal prototype has been tested in a feasibility study. Preliminary results from this study indicate that SmartJournal is well accepted among its users. According to survey data collected from caregivers employed at 3 nursing homes in Rogaland, Norway (n=86), 72% (62/86) reported that SmartJournal is a useful tool, 94% (81/86) reported that it is easy to use, and 69% (59/86) reported that they would like to use a refined version of the tool in the future. Further results from the feasibility study will be published elsewhere.

### Further Improvement of the SmartJournal

Recently, the SmartJournal prototype has been further improved and adapted to the nursing home setting. Since user involvement is a central feature in the development of interventions [32], this improvement was guided by results obtained from the feasibility study (ie, information acquired from meetings, qualitative interviews, and survey questionnaires). The improved version has been subjected to 2 functionality tests in a controlled laboratory environment. These tests were performed by the Norwegian Smart Care Cluster, a national and international collaboration arena for companies, municipalities, hospitals, public organizations, user organizations, academia, research and development institutions, and investors that works to develop sustainable health solutions for the future [33]. Insights gained through the tests have been used for additional, minor adjustments to the tool, which is now ready to be tested in the evaluation study described in the following sections.

### Aims and Objectives

Since SmartJournal seems to be well accepted among its potential users, this study aims to evaluate the impacts of its use. More specifically, the objectives of the current research are to assess (1) the effectiveness of SmartJournal use in enhancing nursing home caregivers’ capability, opportunity, motivation, and routines related to oral care tasks and (2) whether SmartJournal use may result in improved oral health for nursing home residents.

## Methods

### Design

A pragmatic cluster randomized controlled trial [34] will be used to assess the effects of SmartJournal use as specified under study objectives. Public nursing homes located in the southern part of Rogaland, Norway, will be recruited and randomly assigned to an intervention group and a control group. Institutions housing long-term residents will be included, while institutions with primarily short-term residents, institutions already following a specific protocol for oral care, or institutions participating in other comprehensive interventions are excluded (Textbox 1). Nursing homes in the intervention group will be provided with smart tablets containing SmartJournal and user support (a help desk) by research staff employed at the Oral Health Centre of Expertise Rogaland, while nursing homes in the control group will continue with existing oral care routines. Both groups will receive oral hygiene packages, including toothbrushes for permanent teeth and prosthetic devices, dental floss, interdental brushes, toothpaste, and inspection mirrors, to make sure that oral hygiene equipment is easily accessible for all participants. Nursing homes and individual participants will not receive information about who is included in the intervention and control groups, respectively. They will only receive information that is relevant to their participation. The chosen study design (a cluster randomized controlled trial) facilitates this masking by reducing the possibility of contamination between the groups. The intervention will last for 12 weeks. Measurements will be performed at baseline, 3 months, and 9 months.

**Inclusion and exclusion criteria for the SmartJournal evaluation study.**

**Inclusion criteria**

- Nursing homes
  - Located in Rogaland, Norway
  - Long-term facilities
- Residents
  - Long-term residents (ie, residents with the nursing home as their permanent address)
  - Residents with and without dementia who can undergo oral examination
- Caregivers
  - All types of nursing staff (nurses, auxiliary nurses, students)
  - Full-time and part-time staff

**Exclusion criteria**

- Nursing homes
  - Specialized nursing homes (eg, psychiatry and substance abuse)
  - Short-term facilities (eg, rehabilitation and respite care)
  - Nursing homes already following a specific protocol for oral care or those participating in other comprehensive interventions
- Residents
  - Short-term residents
  - Critically ill and terminal patients receiving palliative care
- Caregivers
  - Staff with purely administrative tasks (eg, heads of institutions)

### Participants and Procedures

Strategies used for recruitment of individual participants (caregivers and residents) and procedures for delivery of the intervention will be similar to those used in the feasibility study since they proved to be successful. Resource groups, comprised of heads of wards and selected nursing staff, will be established at each of the included nursing homes. Meetings will be scheduled to adapt the delivery of the project to each individual institution’s resources and facilities, and members of the resource groups will be assigned the task of recruiting individual participants. Written informed consent will be requested for both residents and caregivers to be enrolled in the study. Nursing home residents constitute a vulnerable group in which many individuals may have reduced decision-making capacity. For these individuals, deputy consent will be obtained from their next of kin. Residents receiving end-of-life palliative care are excluded (Textbox 1). Both full-time and part-time nursing staff will be included. Health personnel with purely administrative tasks (ie, heads of institutions) are excluded (Textbox 1). Following recruitment of participants and baseline measurements, nursing homes in the intervention group will be provided with SmartJournal toolboxes and packages with oral hygiene equipment, while nursing homes in the control group will receive oral hygiene equipment packages only. The SmartJournal toolboxes include smart tablets with SmartJournal installed, antibacterial touchscreen wipes, flashlights for monthly oral inspections, an instruction video, an instruction book, and SmartJournal information posters. The oral hygiene equipment packages include toothbrushes for permanent teeth and prosthetic devices, dental floss, interdental brushes, toothpaste, and inspection mirrors. Training, follow-up, and technical support for SmartJournal users will be provided by project staff employed at the Oral Health Centre of Expertise Rogaland.

### Measurements

The effectiveness of SmartJournal in enhancing nursing home caregivers’ capability, opportunity, motivation, and routines related to oral care tasks will be measured by a survey questionnaire capturing components of the COM-B model [27]. Caregivers in both the intervention group and the control group will be asked to fill out the survey questionnaire three times: at baseline, 3 months, and 9 months.

The effects of SmartJournal use on nursing home residents’ oral health will be assessed by low-invasive oral inspections using the mucosal-plaque score (MPS) [17] as a primary outcome measure. MPS is comprised of a 4-point plaque score (PS) and a 4-point mucosal score (MS). PS=1: no visible plaque; PS=2: plaque is barely visible; PS=3: moderate amount of plaque; and PS=4: large amount of plaque almost covering the whole surface of teeth. MS=1: normal mucosa; MS=2: mild inflammation; MS=3: medium inflammation; and MS=4: strong inflammation. For both scores, if in doubt between 1 and 2, dental personnel are instructed to score 1. If in doubt between 3 and 4, they are instructed to score 4. The MPS index is designed to evaluate oral health and oral hygiene in groups of individuals. This makes it a suitable index to be used when conditions are not optimal to assess the patient’s oral health. In a nursing home setting, dental personnel have limited equipment and poor lighting compared to an ordinary dental clinic setting. Therefore, using an index designed to be applied outside a dental clinic setting is feasible in this context. Moreover, the number of natural teeth will be counted and used as an additional outcome measure. The number of natural teeth is a frequently used measure of oral health in older adults since it is associated with nutritional status, general health, and quality of life [28]. Residents in both the intervention group and the control group will have MPS assessed and teeth counted three times: at baseline, 3 months, and 9 months. All clinical assessments will be performed by dental professionals employed at the Oral Health Centre of Expertise Rogaland, Norway. Calibration has been undertaken to ensure the reliability of the clinical measurements.

Additionally, patient journal data shown to be associated with oral health (ie, sociodemographics, medical conditions, and prescribed medication) will be retrieved and used to describe the study sample. These data will also be included as covariates in statistical analyses investigating factors associated with oral health among residents included in this study.

### Power Calculations and Estimation of Sample Size

Experiences from the feasibility study show that resident participation may pose a challenge due to the high rates of morbidity, mortality, and CRB in this part of the population. The estimation of sample size is largely reproduced from Overgaard et al [35], who also used MPS as a primary outcome variable in their study on oral health in nursing home residents. Overgaard et al [35] based their power calculations on reductions in MPS ≥2 from 60% to 15%, as reported by Hede et al [17]. Like Overgaard et al [35], we assume α=.5, a power of 80%, a 33% dropout rate due to the residents’ frailty, and a median resident time of 2 years. A medical statistician has been consulted to further adapt the calculations to this study’s setting (taking into account the number of eligible nursing homes and residents in the southern part of Rogaland), recommending the inclusion of 12 nursing homes each with 30 residents who opted for oral health assessments. Thus, a total of 360 residents will be included for oral health assessments, 180 in the intervention and control groups, respectively. All eligible caregivers in the 12 included nursing homes will be invited to take part in the survey capturing components of the COM-B model as described in the Measurements section. Experiences from the feasibility study indicate that this broad inclusion strategy, along with assigning the recruitment task to members of the institutions’ resource groups, fosters a sense of community regarding project participation. This may, in turn, motivate institutions and individual participants to remain part of the project throughout the study period.

### Data Analyses

The statistical software package SPSS (IBM) will be used for data analyses. These analyses will include descriptive statistics (to describe the samples) and statistical techniques to look for changes and differences in the study variables within and between the intervention group and the control group throughout the project period, for example, 2-tailed *t* tests and ANOVA tests. We hypothesize that SmartJournal use will lead to favorable changes in variable scores in the intervention group as compared to the control group. In caregivers, it will lead to increased scores on the measured COM-B components (survey data), and in residents, it will lead to improved oral health as measured by improved MPS and, possibly, fewer natural teeth lost. Additionally, regression analysis may be used to investigate associations between study variables while adjusting for potential covariates (eg, age, gender, ethnic origin, educational level, medical conditions, and prescribed medication).

### Ethics Approval

This study is approved by the Norwegian Regional Committee for Medical and Health Research Ethics (REK 2022/472796). The SmartJournal intervention aims to assist nondental care providers with preserving nursing home residents’ statutory right to good oral hygiene and necessary dental treatment. Respect for the individual’s intrinsic value, self-determination, and situation pervades the development and anticipated use of the tool. The project is guided by the intentions of the Declaration of Helsinki and ethical guidelines provided by the Norwegian National Research Ethics Committees. Informed consent will be obtained from nursing home caregivers and residents before data collection. Consent forms are designed in accordance with the templates for information material provided by Sikt, the Norwegian Agency for Shared Services in Education and Research, and the Regional Committees for Medical and Health Research Ethics. Nursing home residents constitute a vulnerable group in which many individuals may have reduced decision-making capacity and, thereby, difficulties giving free and informed consent to being participants in research. However, since the overall aim of the SmartJournal intervention is to assist health personnel with preserving oral health in this particular group, it is essential to include them in the research. Therefore, if residents have reduced decision-making capacity, deputy consent will be obtained from their next of kin.

Data registered in SmartJournal (ie, data for residents included in the intervention group) will be transferred to a research database at the University of Stavanger. At the SmartJournal login website, health personnel are asked to enter three codes: one code for the nursing home, one (personal) caregiver code, and one (personal) resident code. The codes for caregivers and residents will be listed in an identification key that is only available to authorized project staff. At the end of the project, data will be deidentified, and it will not be possible for anyone to link any information to individual participants. Data obtained from questionnaires, clinical assessments (MPS, number of teeth), and patient journals are registered with the same individual codes as data registered in SmartJournal, which makes it possible to link data obtained from all sources. Extractions from the University of Stavanger database and data from questionnaires will be stored in a highly secured data system at the Oral Health Centre of Expertise Rogaland. Only authorized project staff will have access to the data.

## Results

Recruitment of nursing homes for the intervention and control groups was completed in February 2023. Recruitment of individual participants (ie, nursing home residents and caregivers) is ongoing, and baseline measurements will be performed consecutively. Results from measurements at all time points (ie, baseline, 3 months, and 9 months) are expected to be ready for analyses in fall 2024.

## Discussion

### Principal Considerations

Poor oral health in care-dependent older adults is a substantial public health problem, as it is associated with reduced general health and quality of life in this growing part of the population [1,2,6]. Research has revealed that oral health is the most neglected area in Norwegian nursing homes [3], resulting in unacceptable oral hygiene in large proportions of nursing home residents [5]. As described in the Background section, numerous barriers to adequate oral care in nursing homes have been reported, including a lack of knowledge and skills among nursing staff, CRB in patients with dementia, a lack of adequate oral care routines and systems for documentation of oral care issues, a high workload, and unclear responsibilities [18-21]. Thus, there is an urgent need for interventions that can improve oral care and oral health among care-dependent older adults. Previous tools developed to assist health personnel in providing oral care to frail older adults have shortcomings, and some of them are not well suited for nondental professionals [24,25]. The SmartJournal intervention was developed to overcome these challenges, following principles of responsible research and innovation in terms of stakeholder involvement, acceptability, functionality testing, and quality testing, with continuous attention to issues related to ethics and sustainability in this study’s context [36].

### Strengths and Limitations

Among the strengths of this study is the application of an established framework for research on complex interventions [26], including theory such as the COM-B model [27], to guide intervention development, testing, and analyses. Another strength is that the study includes data from several different sources and modes of data collection, that is, caregivers’ responses to survey questionnaires, SmartJournal registrations on oral care routines, patients’ oral health status (ie, clinical outcome measures), and selected data from patient journals. Thus, the “common methods problem” is reduced compared to situations where only one data source is available. However, this study also has some limitations that have to be mentioned. The self-report measures used to assess caregivers’ capability, opportunity, motivation, and routines related to oral care may be subjected to bias related to recall, introspective ability (ie, participants may not be able to assess themselves accurately), interpretation of questionnaire items, socially desirable responding (ie, the tendency to answer questions in a manner that will be viewed favorably by others), and sampling (ie, people who complete the questionnaire are the sort of people who are inclined to complete a questionnaire) [37]. There is also a risk of getting a low response rate from caregivers due to the general lack of time and high workload among health personnel [38].

### Conclusions and Dissemination

This study aims to assess the effects of SmartJournal use on oral care and oral health in nursing homes. Results from this work may be used to inform the continued development and implementation of the SmartJournal intervention in nursing homes and other caring facilities, including in-home care. Similarly, the findings may inform the development and implementation of other digital tools aiming to enhance health care services offered to care-dependent people. Moreover, since oral health knowledge and skills seem to be inadequate for nondental health care professionals, SmartJournal may have potential as a learning tool in educational programs for nurses and other health care professionals. Results from the study will be published in high-impact academic journals and other relevant channels of dissemination, including academic and practitioners’ conferences and various public media.

### Peer Review

Multimedia Appendix 1 contains the peer review report by the Regional Research Funds Rogaland, Norway.

### Time Schedule

The study is scheduled between February 1, 2023, and December 31, 2025 (Table 2).

**Table 2:** Anticipated time schedule and research activities for the SmartJournal evaluation study.

Activities	Period
Recruitment and baseline measurements (T0)	First quarter of 2023 to second quarter of 2023
Intervention^a^	Second quarter of 2023 to fourth quarter of 2023
Measurements at 3 months (T1)^a^	Fourth quarter of 2023 to first quarter of 2024
Measurements at 9 months (T2)^a^	First quarter of 2024 to third quarter of 2024
Analyses and dissemination	Fourth quarter of 2024 to fourth quarter of 2025

^a^The intervention will last for 12 weeks. Since the start date may vary between the included nursing homes, a 12-month period is scheduled to perform the intervention and measurements at T0, T1, and T2.

## Appendix

Multimedia Appendix 1 Peer review report by the Regional Research Funds Rogaland, Norway (Hovedprosjekt Regionalt forskningsfond Rogaland - vedtaksbrev - 332666).

## Abbreviations

COM-B: capability, opportunity, motivation model of behavior
CRB: care-resistant behavior
e-learning: electronic learning
MPS: mucosal-plaque score
MS: mucosal score
PS: plaque score
ROAG: Revised Oral Assessment Guide

## Data Availability

The data sets generated and/or analyzed during this study are not publicly available due to the sensitive nature of the information, and because participants’ consents for public data sharing were not obtained.
