# Supplementary material for: Assessment of the SmartJournal Intervention for Improved Oral Care in Nursing Homes: Protocol for a Cluster Randomized Controlled Trial
Source: JMIR Res Protoc. 2023 Oct 6;12:e46926. doi: 10.2196/46926 (PMC10589829; doi:10.2196/46926)
Supplement: Multimedia Appendix 1 [file resprot_v12i1e46926_app1.pdf]

REGIONALE FORSKNINGSFOND ROGALAND

VEDTAK – Regionalt forskningsfond Rogaland (Støtte fra andre enn Forskningsrådet)

**PROGRAM:**	RFFROGALAND
**UTLYSING:**	Regionalt innovasjonsprosjekt for offentlig sektor. (Søknadsfrist 20.10.2021)
**PROSJEKTNUMMER:**	332666
**PROSJEKTTITTEL:**	SmartJournal for oral helse i sykehjem
**SØKER:**	TANNHELSE ROGALAND FKF
**SAMARBEIDSPARTNERE:**	UNIVERSITETET I STAVANGER, UNIVERSITETET I AGDER, Utviklingssenter for sykehjem og hjemmetjenester (USHT), Norwegian Smart Care Cluster

SØKT BELØP (i hele 1000 kr): 3 000

**KOSTNADSPLAN (i hele 1000 kr)**		**FINANSIERINGSPLAN (i heile 1000 kr)**	
Personal- og indirekte kostnader:	8 666	Eigne midler	6 829
Innkjøp av FoU-tjenester:	700	Internasjonale midler	0
Utstyr:	0	Andre offentlige midler	0
Andre driftskostnader:	463	Andre private midler	0
		Søkes Regionale forskingsfond	3 000
Totalsum:	9 829	Totalsum:	9 829

HOVEDMÅL OG DELMÅL

Prosjektets hovedmål er å kvalitetssikre og verifisere effekten av et digitalt verktøy, SmartJournal, som skal bistå pleiepersonell med å ivareta sykehjemspasienters munnhelse, og dermed legge til rette for redusert sykdomsbelastning og bedre livskvalitet hos denne sårbare gruppen av befolkningen. Prototypen av SmartJournal er allerede utviklet og testet i en mulighetsstudie.

Prosjektets hovedmål skal realiseres gjennom følgende delmål:

1. Optimalisere/oppdatere SmartJournal basert på resultater fra mulighetsstudien og testing av verktøyet i Smart Care Cluster Lab.
2. Verifisere nytten/effekten av SmartJournal i en intervensjonsstudie med kausalt design i sykehjem.

Når delmål 1 og 2 er gjennomført, skal verktøyet frigjøres for bruk i generell praksis og en evt. kommersialisering.

PROSJEKTSAMMENDRAG

Forskning viser at det er store utfordringer knyttet til oral helse hos pleietrendende eldre. Mange sykehjemspasienter har dårlig oral hygiene, og munnstell er den oppgaven som først blir nedprioritert ved mangel på ressurser. Dårlig oral hygiene gir økt risiko for orale og systemiske infeksjoner og sykdommer. Dårlig oral helse er også forbundet med underernæring og redusert livskvalitet. Tidligere hadde de fleste eldre gebiss. I dag beholder stadig flere egne tenner livet ut, ofte i kombinasjon med kostbare tannerstatningsløsninger som broer, kroner og implantater. Disse løsningene krever en annen grad av renhold og oppfølging enn et gebiss som er enkelt å ta ut og rengjøre. Det er en utfordring at helsepersonell mangler tilstrekkelig kunnskap om oral helse og riktig munnstell. Særlig gjelder dette stell av ulike tannerstatningsløsninger. Det er også en utfordring at mange pasienter, spesielt personer med demens, motsetter seg hjelp til munnstell. I tillegg er det mangel på gode rutiner og systemer for oppfølging og registrering av avvik relatert til oral helse i sykehjem. Dette prosjektet skal bidra til å løse disse utfordringene ved å kvalitetssikre og verifisere nytte/effekter av det digitale lærings- og dokumentasjonsverktøyet, Smartjournal (SJ), som er utviklet for å hjelpe pleiepersonell med å ivareta sykehjemspasienters orale helse på forsvarlig vis. Utvikling og testing av en SJ prototype i sykehjem er allerede gjennomført. Forskningen i prosjektet det nå søkes midler til inkluderer optimalisering og laboratorietesting av SJ, samt verifisering av verktøyet i en intervensjonsstudie med kausalt design. Dette arbeidet skal gjennomføres i samarbeid med regionale og nasjonale FoU-miljøer med spesialistkompetanse innen odontologi, geriatri,

helseteknologi og helserelatert intervensjonsforskning. For å sikre god kvalitet og brukermedvirkning i alle ledd av forskningen, baseres arbeidet på UK Medical Research Council's (MRC) rammeverk for komplekse intervensjoner.

VURDERING

Kriterium	Karakter	Kommentar*
Forskning og innovasjon	5	Prosjektet representerer noe nytt og løser en utfordring med et erkjent, godt dokumentert behov, og prosjektet er godt knyttet til behovene i sykehjemmene. Prosjektet har et stort potensial for å produsere ny kunnskap som er svært viktig for at innovasjonen skal lykkes og bli relevant. Innovasjonen har stor mulighet til å løse utfordringen som er beskrevet i søknaden.<br><br>Det vurderes at FoU-aktivitetene i er nødvendige for å lykkes med innovasjonen. Prosjektet bruker oppdatert kunnskap og relevante FoU-metoder og prosjektet tar utgangspunkt i relevante forskningsartikler som underbygger problemstilling. Det vurderes imidlertid at søknaden mangler mål på endring av oral hygiene.
Virkninger og effekter	6	Søknaden har troverdige og tydelig formulerte virkninger og effekter, med viktige samfunnseffekter som bidrar til FNs bærekraftsmål, og bedret kvalitet i tjenesten. Det er lagt opp til baseline målinger og systematiske målinger av virkninger og effekter. Det vurderes som positivt at den potensielle effekten er kvantitativt estimert.<br><br>Prosjektet legger opp til god planlegging med aktuelle samarbeidspartnere og meget god forankring hos helsepersonell.<br><br>Det vurderes at målet om bedre tannhelse er mangelfullt beskrevet.<br><br>Søknaden beskriver meget gode kommunikasjons- og kommunikasjonsaktiviteter til riktige målgrupper, med en tydelig og sannsynliggjort plan for formidling bl.a. i vitenskapelige artikler og til tjenesteytere.
Gjennomføring	6	Prosjektplan fremstår fornuftig og gir godt grunnlag for FoU-aktivitetene og nyttegjøring av resultat. Meget god oppfølging og felles aktiviteter i prosjektgruppa. Positivt at prosjektet deltar i GERONETT aktiviteter, samt god brukermedvirkning i Utviklingssenter for sykehjem og hjemmetjenester (USHT). Miljøet bygger på MRC rammeverket, en state-of-the art metode for helsetjenesteutvikling. Søknaden har en god beskrivelse av roller av ansvar.<br><br>Solid prosjektorganisering - prosjektledelse og prosjekteierskap/forankring. Prosjektgruppen har relevant erfaring og kompetanse. Arbeidet inkluderer aktiv deltagelse fra sykehjem og fagekspertise på oral helse. Det er imidlertid mangelfullt beskrevet i søknaden hvordan ansvar for gjennomføring skal gjennomføres på avdelingsnivå på sykehjemmene. Erfaringsmessig er dette veldig viktig for deltagelse og gjennomføring.<br><br>Milepælsplanen er godt knyttet til budsjett og aktiviteter.<br><br>Det vurderes at risikovurderingene er noe mangelfull.
Samlet vurdering fra fageksperten/panelet	6	Søknaden svarer veldig godt til kriteriet. Innovasjonen frembringer ny kunnskap og møter et tydelig og veldokumentert behov. Tydelig beskrivelse av innovasjonen, og prosjektet er avgrenset til konkrete utfordringer og løsninger. Prosjektet sin gjennomføring er beskrevet på en god og realistisk måte med tydelig rollefordeling.<br><br>God kompetanse i prosjektorganisasjon. Brukermedvirkning som er viktig for å bidra med resultater. God forankring hos samarbeidsparter. God fagkunnskap med regionale og nasjonale

		FoU-miljøer med spesialistkompetanse innen odontologi, geriatri, helseteknologi og helserelatert intervensjonsforskning. God struktur på prosjektet og godt potensial for effekt og verdiskaping.<br><br>Prosjektet vurderes å ha noen mangler i beskrivelse av endringsmål i oral hygiene hos beboere, og forankring og involvering på avdelingsnivå.

7: Utmerket, 6: Svært godt, 5: Mye godt, 4: Godt, 3: Mindre godt, 2: Svakt, 1: Dårlig

** RFF Rogaland gjør oppmerksom på at kommentarene fra ekspertene ikke representerer hele diskusjonen rundt søknaden. Kommentarene er ført i pennen av administrasjonen, og er et sammendrag av ekspertenes vurdering av hvert kriterium. Vi håper prosjektansvarlig kan bruke kommentarene som innspill i det videre arbeidet med prosjektet.*

VEDTAK:

Regionalt forskningsfond Rogaland gir tilsagn om inntil kr 3 000 000 (inntil 50 % av godkjente prosjektkostnader) i støtte til:

PROSJEKT: 332666 SmartJournal for oral helse i sykehjem
PROSJEKTANSVARLIG SØKER: TANNHELSE ROGALAND FKF

DATO FOR VEDTAK: 08.02.2022

Sekretariatet i Regionalt forskningsfond Rogaland
